# Behavioural Profile Differences Between Cats in Animal-Assisted Services (AAS) and Non-AAS Cats Using the Fe-BARQ in Flanders

**DOI:** 10.3390/ani15010033

**Published:** 2024-12-26

**Authors:** Joni Delanoeije, Christel Palmyre Henri Moons, Els Helena Karel Anna Peeters, Patricia Pendry

**Affiliations:** 1 Work and Organisation Studies, Faculty of Economics and Business, KU Leuven, Hendrik Conscienceplein 8 Box 3530, 2000 Antwerpen, Belgium; 2 Research Foundation Flanders (FWO Vlaanderen), Egmontstraat 5, 1000 Brussels, Belgium; 3 Ethology and Animal Welfare Research Group, Department of Veterinary and Biosciences, Faculty of Veterinary Medicine, Ghent University, Heidestraat 19, 9820 Merelbeke, Belgium; christel.moons@ugent.be; 4 SALTO Research Group Agro- and Biotechnology, Odisee University of Applied Sciences, Hospitaalstraat 23, 9100 Sint-Niklaas, Belgium; els.peeters@odisee.be; 5 Human Development, College of Agricultural, Human and Natural Resource Sciences, Washington State University, Pullman, WA 99164, USA; ppendry@wsu.edu

**Keywords:** feline behaviour profile, animal-assisted services, animal-assisted interventions, domestic cat, cat behaviour, Fe-BARQ, questionnaire

## Abstract

The engagement of cats in animal-assisted services (AAS) is increasing. This is surprising given that feline behavioural needs have been perceived as contradictory to conditions associated with AAS engagement, leading to the assumption that cats as a species are not suitable for AAS. However, important within-species variability in behavioural traits nuancing this assumption has not been addressed. Therefore, this study aimed to map the behavioural profiles of cats engaged in AAS and non-AAS cats to detect behavioural profile differences between these groups. Such differences could point to AAS cats being selected to engage in AAS based upon particular behavioural traits or, relatedly, to AAS cats coping differently with AAS features. Using a Flemish translation of the Feline Behavioural Assessment and Research Questionnaire (Fe-BARQ), cross-sectional survey data on various cats’ (*N* = 474) behavioural indices were collected. Using Mann–Whitney U-tests, results indicate that AAS cats significantly differed from non-AAS cats on five out of 19 behavioural subscales. Specifically, AAS cats scored higher on sociability with people, attention seeking, sociability with cats and predatory behaviour, and they scored lower on resistance to restraint. While this study did not allow for assessing explanations for these differences, findings suggest that cats in AAS may be intentionally or unintentionally selected for behavioural traits perceived as desirable for engagement in AAS. While we refrain from drawing conclusions regarding the “suitability” of cats based solely on our findings, our study contributes to the ongoing discourse surrounding critical themes around AAS-engaging cats. Furthermore, our findings provide a first indication of the reliability of a translation of a well-validated measurement tool suitable to map behavioural traits of cats in Flanders engaged in AAS.

## Simple Summary

Cats are increasingly involved in animal-assisted services (AAS), which help people with physical, emotional, or mental challenges. However, some believe cats may not be suitable for this work because of their species-specific needs and behaviours. This study explored whether cats participating in AAS have different behavioural traits compared to other cats. Using a Flemish translation of the Feline Behavioural Assessment and Research Questionnaire (Fe-BARQ) completed by 474 cat caregivers in Flanders, we found that AAS cats scored higher on sociability with people, attention seeking, sociability with cats, and predatory behaviour, and they scored lower on resistance to restraint, compared to other cats. These findings suggest that cats chosen for AAS might naturally exhibit traits that help them thrive in this role. While this study does not make conclusions about whether cats as a species are suitable for AAS (i.e., focusing on between-species differences), it adds to our understanding of how cats might support humans through AAS, taking into account individual characteristics (i.e., focusing on within-species differences). Furthermore, this research tested a useful tool to study cat behaviour, helping to improve how we assess the engagement of cats in AAS now and in the future.

## 1. Introduction

The engagement of cats in animal-assisted services (AAS) is increasing [1]. Previously known as animal assisted interventions (AAI) [2], the most recent definition of AAS includes “services that are facilitated, guided or mediated by a health or human service provider or educator, who works with and maintains the welfare of a specially qualifying animal to provide therapeutic, educational, supportive, and/or ameliorative processes aimed at enhancing the well-being of humans” [3] (p. 1). While dogs are the most common animal species engaged in AAS [4,5], recent studies have described the gradually increasing engagement of cats [6,7,8,9] (for a literature review, see [1]). This may seem surprising given that feline behavioural needs (e.g., predictability of social interactions, stable environment) may appear contradictory to the conditions associated with AAS settings (e.g., crowding, repeated relocation; IAHAIO, 2018), with some suggesting that cats as a species are “unsuitable” for AAS. However, this assumption does not consider important within-species variability in feline behavioural traits. This is surprising, as behavioural differences between cats may suggest variance in cats’ enjoyment of and their coping with features associated with AAS. Additionally, it has been suggested that cats engage in behaviours that make them unsuitable for AAS. However, like dogs, cats have been found to be trainable, to be responsive to human communicative clues, to share close relationships with people, and to trigger physiological and psychological effects in humans [10,11]—characteristics which have been described as desirable for dogs engaging in AAS [12,13].

While the AAS literature contains references to preferred behaviour traits of animals engaged in AAS, the empirical evidence supporting consistent differences in behaviour profiles is mixed. Specifically, in dogs, previous work has shown that high sociability and reduced fear and aggression are desirable behavioural traits for engagement in AAS [14,15,16]. Research has found that dogs engaged in AAS (“AAS dogs”) were less impulsive and showed increased gazing toward humans as measured by the Dog Impulsivity Assessment Scale (DIAS [17]) and a gazing task [12] compared to dogs who did not have experience participating in AAS (“non-AAS dogs”). Yet, no differences in behavioural traits were found between AAS dogs and non-AAS dogs when dogs were assessed using subscales of the Canine Behavioural Assessment and Research Questionnaire (C-BARQ [18]). Specifically, the C-BARQ subscales of trainability, fear of strangers, non-social fear, and attachment/attention seeking of the caregiver were not different between both groups of dogs [12]. Another study showed that AAS dogs looked more at their caregiver during an unsolvable task, which also suggests enhanced attention of these dogs toward their caregiver as compared to non-AAS dogs [13].

While interesting, the mixed findings on behavioural profile differences in AAS and non-AAS dogs may reflect both the measurement variability as well as the variety of used definitions of AAS. Therefore, the use of uniform measurement across situations is paramount in order to understand potential differences in animals engaged in AAS compared to other animals. The limitations of current measurement approaches are especially relevant for comparing behavioural profiles of AAS and non-AAS cats, about which virtually nothing is known. In fact, despite the increasing prevalence of cats engaged in AAS, their population is relatively small and dispersed, which has provided a substantive challenge to researchers interested in studying their behaviour, contributing to a significant gap of knowledge about them.

Describing and comparing the behavioural traits of cats that engage in AAS (“AAS cats”) with those who do not is important in three main ways. First, considering the context of increasing engagement in AAS, it may help understanding selection effects concerning feline AAS; that is, understanding why cats with certain behavioural traits are selected by people for engagement in AAS. Understanding which behaviour traits appear to be more prevalent in AAS populations may exemplify people’s preference for certain behavioural profiles in AAS cats. Ultimately, these insights may contribute to our understanding of the mechanisms responsible for the therapeutic effects of feline AAS [19,20]. Specifically, if cats with certain behavioural traits are overrepresented in AAS practices, this may point towards preferred traits for AAS to be effective in terms of cats’ favourable impact on human well-being [14,20,21], and/or traits most welcomed by people [7].

Second, like for dogs [19], feline behavioural profile scales could be used to identify key behavioural patterns of cats useful for selection and well-being evaluation of AAS cats. Whereas we consider the use of caregiver-rated questionnaires for determining the suitability of cats for AAS practices to be limited, insights into cat’s individual behavioural profile scores and group ratings might function as a convenient method for AAS organisations to gather information about prospective AAS cats in a systemic way with respect to the cat’s well-being. Specifically, if AAS cats exhibit behavioural traits different from non-AAS cats, this might indicate that AAS cats cope differently with situations and potential stressors compared to non-AAS cats, as behavioural characteristics may be associated with ways of coping with stress. This may ultimately have implications for cat well-being [22], as this may suggest that AAS cats may experience features of AAS (e.g., interaction with people; repeated relocation) differently. That said, these potential implications are not considered particularly relevant for immediate application given the exploratory nature of this research and the likely limited generalizability of our sample of AAS cats.

Third and last, insights in behavioural profiles of AAS cats using uniform terminology and measurement help the standardization of used protocols and their description in AAS. Such standardization is paramount to better understand process and outcome evaluation of AAS and to monitor cat well-being [20,23,24]. Just as is the case for dogs, having insights into behavioural profile differences between cats that are engaged by people in AAS (“AAS cats”) and cats that do not participate in such practices (“non-AAS cats”) using uniform and updated AAS terminology [3,25,26,27] and standardised behavioural profile measurement may further our understanding of feline AAS.

The main aim of the current study is to map the behavioural profiles of AAS cats and non-AAS cats to assess differences in behavioural profiles between groups using a reliable, well-validated measurement tool and standardised terminology of AAS. To achieve this aim, we collected cat behavioural profile data using the Feline Behavioural Assessment and Research Questionnaire (Fe-BARQ [28,29]), which is the most-used questionnaire to assess behavioural profiles in cats. We translated the measure into Flemish and compared scores on 23 behavioural indices between AAS cats and non-AAS cats in Flanders. Cats were considered as AAS cats in case their caregiver indicated to engage in at least one of the five categories of AAS with their cat, using category definitions as provided by the International Association of Human-Animal Interaction Organizations [2] (pp. 5–6). Due to the expected challenge of identifying and recruiting an adequate number of AAS cats, we employed statistical tools designed specifically to conduct reliable statistical comparisons between populations featuring different sample sizes.

While our study is the first to explore behavioural profile differences between these groups of cats, our study is not designed to determine the suitability of cats for AAS but rather serves as a first exploration of whether differences in behavioural profiles exist in cats who are actually engaged in AAS compared to cats who are not. Specifically, we hypothesised that cats in our sample that were engaged in AAS would differ on behavioural indices indicative of behaviours desirable for AAS, including playfulness/activity, sociability, attention-seeking behaviour, stranger-directed aggression, resistance to restraint, fear of novelty, trainability, and location preference for resting/sleeping.

## 2. Materials and Methods

The study was conducted in Flanders, Belgium, and comprised an online anonymous survey targeted to cat caregivers. Since the study did not engage with cats directly or engage in manipulation of conditions, the study is not considered an animal experiment. The study was exempt from full board review since we collected data using an electronic survey without inquiring about and/or collecting any personal and/or identifiable data. All participants were consented and electronically signed an informed consent form before completion of survey data.

### 2.1. Recruitment, Design and Sample

In 2021, using the online Qualtrics platform, we conducted the survey in which cat behaviour profile data were collected from cat caregivers. Invitations to participate were distributed online through convenience sampling targeting cat caregivers through various channels. The recruitment flyer described a call for “participation in an online study about cat personality, with a special interest in cats engaged in AAS or cats who accompany their caregivers outside their residence” and included a picture of a cat.

The first part of the survey included questions on demographics and background features of the cat, including the cat’s participation in joining the caregiver during outings outside of their residence and in the cat’s engagement in AAS (formerly known as Animal Assisted Interventions, or AAI) as defined by the former terminology of AAI by IAHAIO [2]. The second part of the survey included our Flemish translation of the Fe-BARQ [28], measuring 23 behavioural indices using 85 items. Last, the third part included questions on demographics and background features of the cat caregiver (i.e., gender, age, and education level).

Respondents were included if they lived in Flanders and had a cat aged one year or older that had been living with the respondent for a minimum of 6 months. Furthermore, each respondent could only fill out the survey once, so it was not possible to answer the survey for multiple cats. If respondents indicated they had more than one cat at home, they were asked to choose one focal cat—if applicable, the cat that either joins the caregiver during outings or is engaged in AAS. In total, we collected complete survey data on 474 cats. Respondents indicated having one to 19 cats at home (*M* = 2.1; *SD* = 1.85), and 55.9% had a multi-cat household, with 44.1% having one cat, 33.8% having two cats, 12.2% having three cats, and 9.9% having four cats or more. Cats were between one and 21 years old (*M* = 6.2; *SD* = 4.2) and resided between one and 20 years with their caregiver (*M* = 5.4; *SD* = 4.0). Most of the cats in the sample were female (51.1%), were neutered (98.1%), and did not have a pedigree (78.9%). Cat caregivers were mainly female (90.7%) and were between 16 and 78 years old (*M* = 36.3; *SD* = 11.7). Caregiver education was reported to be 40.9% higher education short type (BSc), 24.7% higher education long type (MSc), 23.9% high school, and 4.2% PhD.

### 2.2. Flemish Translation of the Fe-BARQ

The instrument to measure cat behavioural profiles included a Flemish translation of the Fe-BARQ questionnaire [28]. After having obtained permission from the first author of this questionnaire, two of the current authors (JD, CM), native Flemish speakers, translated the questionnaire to Flemish, and a third author (PP), native and fluent in both languages, back-translated this version to English [30]. Discrepancies between the translated and the original version were addressed by all authors and a BSc student in Animal Care, considering meaning and appropriateness for identifying cat behaviour. In the case when subtle differences were identified between terms and descriptions in Dutch (spoken in The Netherlands) and Flemish (spoken in Flanders, the northern region of Belgium), the research team consistently applied Flemish terms as the translation language.

### 2.3. Measures

#### 2.3.1. Engagement in AAS

Cat engagement in AAS was determined by means of five categories of Animal Assisted Interventions (AAI)—the former terminology for AAS at the time of data collection—as described by AAI in the IAHAIO white paper [2]. These categories included Animal Assisted Intervention (AAI), Animal Assisted Therapy (AAT), Animal Assisted Education (AAE), Animal Assisted Coaching (AAC), and Animal Assisted Activity (AAA). The definitions for these categories [2] (pp. 5–6) were translated to Flemish by the main researcher. Respondents were asked to identify activities they and their cat participated in during the past two years, and they could indicate multiple categories. In total, 2.1% (*n* = 10) participated in AAI, 1.3% in AAT (*n* = 6), 0.6% in AAA (*n* = 3), 0.4% in AAE (*n* = 2), and 0.4% in AAC (*n* = 2). In total, we coded 2.5% of cats as “AAS cats” (*n* = 12), meaning that their caregiver indicated they participated in at least one category.

#### 2.3.2. Behavioural Subscales

Behavioural indices included the 23 behavioural subscales, covering 85 items in total, from the Fe-BARQ [2], where caregivers rate the frequency of behaviours in their cat. The response scale ranged from 1 (never) to 5 (always) and included a sixth “unknown” option, which was coded accordingly. In line with the original scoring method, a subscale score was coded as missing if more than 25% of the items were missing values. We assessed reliability through calculating Cronbach’s alpha. In total, 19 subscales displayed adequate to high reliability (0.74 < α < 0.95). Four subscales displayed mediocre to bad reliability and were therefore excluded from analysis. These subscales were *excessive/compulsive self-grooming* (α = 0.61), *other compulsive behaviours* (α = 0.60), *crepuscular activity* (α = 0.50), and *location preference resting/sleeping* (α = 0.30). Table 1 shows an overview of the subscales and their reliability indices in the original version of the Fe-BARQ [28] and in our sample.

### 2.4. Statistical Approach

To ensure meaningful comparisons, we pursued sample sizes representative of the respective populations, recognizing the likely smaller population of AAS-engaging cats compared to non-AAS cats [1]. Consequently, our study’s sample sizes differ, reflecting constraints on obtaining AAS-engaging cat samples. Additionally, since we are particularly interested in variation in behavioural traits between groups, we intentionally avoided sample matching to preserve crucial between-group variances and maximise heterogeneity, facilitating meaningful comparisons between the samples.

Accounting for unequal group size and variances of independent groups, we conducted Mann–Whitney U-tests (2-tailed, non-matched samples) to examine group differences based on whether cats engaged in at least one type of AAS, that is, comparing scores of each behavioural subscale between “AAS cats” and “non-AAS cats”. To calculate effect sizes, we used Cohen’s *r* [31]. While unequal and small group sizes may incite concerns about statistical power, the Mann–Whitney U-test is a non-parametric test specifically designed to yield significant results even with unequal sizes, provided that the effect size is sufficiently robust. In fact, this approach is commonly used in scenarios where it is not feasible to obtain equal numbers of participants due to a limited availability of a certain population. Thus, maintaining unequal group sizes, rather than matching sample sizes, can enhance the ecological validity of the findings by providing insights that are applicable to a real-life situation. We also conducted Mann–Whitney U-tests to control for effects of other differences between both groups, testing whether cats in both groups differed from each other on background characteristics.

## 3. Results

We present an overview of our findings in Table 2. Ruling out confounding effects, we found no differences between AAS cats and non-AAS cats concerning cat age (*U* = 2024.50, *Z* = −1.60, *p* = 0.110, *r* = 0.07), cat female sex (*U* = 2256.00, *Z* = −1.27, *p* = 0.203, *r* = 0.06), years resident with caregiver (*U* = 2193.00, *Z* = −0.02, *p* = 0.986, *r* = 0.00), neutering status (*U* = 2589.00, *Z* = −1.65, *p* = 0.098, *r* = 0.08), caregiver female gender (*U* = 2519.50, *Z* = −1.07, *p* = 0.284, *r* = 0.05), or caregiver age (W = 2663.50, *Z* = −0.23, *p* = 0.817, *r* = 0.01).

Assessing our main research question, compared to non-AAS cats (non-matched), AAS cats scored higher on the behavioural subscales *sociability with people* (*U* = 1561.50, *Z* = −1.86, *p* = 0.048, *r* = 0.10), *attention seeking* (*U* = 1825.50, *Z* = −2.029, *p* = 0.042, *r* = 0.09), *sociability with cats* (*U* = 280.50, *Z* = −2.09, *p* = 0.036, *r* = 0.17) and *predatory behaviour* (*U* = 59.50, *Z* = −2.73, *p* = 0.006, *r* = 0.22), and lower on the subscale *resistance to restraint* (*U* = 501.00, *Z* = −2.18, *p* = 0.029, *r* = 0.14). We found no differences on the subscales *playfulness/activity* (*U* = 1890.50, *Z* = −1.86, *p* = 0.064, *r* = 0.09), *directed calls/vocalizations* (*U* = 2394.00, *Z* = −0.31, *p* = 0.759, *r* = 0.01), *purring* (*U* = 2236.00, *Z* = −0.78, *p* = 0.439, *r* = 0.04), *stranger-directed aggression* (*U* = 23020, *Z* = −0.01, *p* = 0.995, *r* = 0.00), *touch sensitivity/caregiver-directed aggression* (*U* = 2158.50, *Z* = −1.35, *p* = 0.178, *r* = 0.06), *familiar cat aggression* (*U* = 909.50, *Z* = −1.75, *p* = 0.080, *r* = 0.11), *dog-directed aggression* (*U* = 365.50, *Z* = −0.27, *p* = 0.788, *r* = 0.02), *fear of unfamiliar dogs/cats* (*U* = 483.00, *Z* = −0.81, *p* = 0.419, *r* = 0.06), *fear of novelty* (*U* = 1979.50, *Z* = −1.11, *p* = 0.266, *r* = 0.05), *separation-related behaviour* (*U* = 1946.50, *Z* = −0.46, *p* = 0.646, *r* = 0.02), *trainability* (*U* = 1910.00, *Z* = −1.09, *p* = 0.276, *r* = 0.05), *prey interest* (*U* = 2048.50, *Z* = −0.47, *p* = 0.636, *r* = 0.02), *inappropriate elimination* (*U* = 2726.00, *Z* = −0.06, *p* = 0.951, *r* = 0.00), and *elimination preferences* (*U* = 1831.00, *Z* = −0.06, *p* = 0.591, *r* = 0.03).

In sum, our findings highlight differences between AAS cats and non-AAS cats on five out of 19 behavioural subscales. Specifically, AAS cats scored higher on *sociability*, *attention seeking*, *sociability with cats*, and *predatory behaviour*, and they scored lower on *resistance to restraint*, as compared to non-AAS cats. These differences were not explained by other cat or caregiver background characteristics included in our measures. Our results are promising as they demonstrate clear differences in behavioural profiles using appropriate statistical testing, despite the smaller sample size relative to the larger population of cats. Such insights are invaluable for practitioners and researchers, providing an initial step towards evaluating cat engagement in AAS.

## 4. Discussion

### 4.1. Behavioural Differences Between AAS Cats and Non-AAS Cats

This study investigated behavioural profile differences between cats engaged in AAS (“AAS cats”) and cats that are not engaged in such practices (“non-AAS cats”), using standardised measurement (i.e., using a Flemish translation of the Fe-BARQ [28]) and standardised terminology (i.e., using the 2018 IAHAIO definitions of Animal Assisted Interventions or AAI, which are currently referred to as Animal Assisted Services or AAS [3,27]). Our findings demonstrated behavioural profile differences on five out of 19 behavioural subscales of the Fe-BARQ, including higher scores on *sociability*, *attention seeking*, *sociability with cats* and *predatory behaviour*, and lower scores on *resistance to restraint* in a sample of 12 AAS cats compared to 462 non-AAS cats. These differences were not explained by group differences based on cat (i.e., cat age, cat sex, years resident with caregiver, neutering status) or caregiver (i.e., caregiver gender, caregiver age) features. Given that employed statistical methods are used to assess significance in groups varying in size, meaningful interpretations of observed differences were made.

Our findings are important for two main reasons. First, they demonstrate behavioural distinctions between AAS and non-AAS cats, as assessed by the Fe-BARQ caregiver-rated questionnaire, paralleling observations in AAS dogs assessed through impulsivity questionnaires [17] and experimental tasks [12,32]. In addition, in our study, significant differences emerged across five Fe-BARQ subscales: *sociability*, *attention seeking*, *sociability with cats*, and *predatory behaviour* (higher in AAS cats), and *resistance to restraint* (lower in AAS cats). The presence of these differences suggests that these behavioural traits may be more desirable (i.e., those on which AAS cats score higher) or less desirable (i.e., the traits on which AAS cats score lower) in AAS cats, or may be associated with specific aspects of AAS, such as heightened social interactions with humans.

Second, our findings illustrate the utility of the Fe-BARQ in detecting behavioural differences between AAS and non-AAS cats, affirming its viability for individual assessment. Specifically, our study demonstrates the internal consistency of a Flemish translation of the Fe-BARQ across 19 out of 23 subscales compared to the original English version [28], with five of these scales distinguishing between AAS and non-AAS cats. This translation facilitates greater application and validation across populations. Individual score assessment is important given the significant behavioural variability observed in cats, similar to dogs [16], indicating that certain cats may be better suited for specific AAS practices, thereby potentially mitigating adverse feline well-being effects.

While our study did not ascertain why these differences were observed, we suggest two possible explanations. First, disparities may stem from pre-existing behavioural variations between AAS and non-AAS cats, possibly reflecting conscious or unconscious selection effects driven by human preferences for specific traits in AAS cats or reflecting cats’ differential coping strategies with AAS-related stressors. Selection effects may indicate human preferences for certain traits in AAS cats, for instance, because the traits are associated with increased engagement in social interactions with people. This suggests implications for the mechanisms underlying the intended benefits of AAS on human well-being, as previous research has linked people’s enthusiasm for AAS and their expectation that the AAS will have beneficial effects—referred to as “program responsiveness” [22,33]—to AAS efficacy. While the influence of program components [8,34,35], longitudinal interaction patterns between people and animals [36], and participant species-specific preferences [7] in shaping AAS participant responsiveness have been investigated, the role of individual cat characteristics remains underexplored. Additionally, observed differences could indicate that cats with certain behavioural traits cope differently with AAS features, suggesting varying levels of resilience or coping strategies. Behavioural traits may be linked to coping styles, defined as “correlated set[s] of individual behavioural and physiological characteristics that [are] consistent over time and across situations” [37] (p. 4021). Therefore, certain traits may be associated with coping styles to manage potential stressors arising from AAS engagement or with individual preferences for social interactions with people. Likewise, some cats may actively enjoy certain AAS features, increasing positive well-being states [38]. Interestingly, AAS cats exhibited significantly higher scores in predatory behaviour compared to non-AAS cats, yet no differences were observed in playfulness. This is particularly noteworthy as caregivers sometimes interpret predatory behaviour as play, suggesting a potential link between the two traits [39]. Potential pre-existing differences in these behaviours are intriguing and warrant further exploration in future research.

Second, our findings may suggest that the engagement of cats in AAS modulates feline behaviour, thus indicating behavioural changes resulting from cats’ engagement in AAS activities rather than reflecting pre-existing behavioural differences. Notably, features inherent in AAS, such as heightened social interactions and potential relocation, may impact feline behaviour. The observed differences in sociability, attention seeking, sociability with cats, predatory behaviour, and resistance to restraint suggest that these traits could be influenced by increased social interactions with people (e.g., through socialization effects [40]), potentially due to learning effects such as reinforcement or habituation. For instance, the lack of reinforcement for expressing certain behaviours may modulate these behaviours [32,41]. Enhanced interactions, particularly with unfamiliar individuals, may especially affect sociability, attention-seeking behaviour, predatory behaviour and resistance to restraint, as cats may repeatedly experience reinforcement of certain attention-seeking and predatory behaviours, and they may habituate to restraint.

### 4.2. Future Research

Our findings demonstrate the feasibility of using the Fe-BARQ to assess individual scores and discern differences between AAS and non-AAS cats. Expanding on these descriptive findings, future research could compare these scores with general ratings to potentially select cats for specific AAS activities. If certain feline behavioural profiles cope differently with AAS-related features, or if AAS engagement modifies cat behaviour, feline behavioural profile scales could aid in identifying key patterns useful for selection and well-being evaluation of AAS cats, similar to current practices in dogs. For instance, in dogs, ratings on the C-BARQ, akin to the Fe-BARQ, inform the selection and training of assistance dogs, predicting training outcomes (e.g., [19]).

Importantly, as evidenced in canine research [16,42], any framework delineating desirable traits for service or working animals must be based on a sound biological framework, given the current inconsistencies in terminology within dog assessments. For dogs, specific tests recommended for assessing desirable traits in various working roles are lacking, prompting scholars to advocate for an initial consideration of the naturally performed (i.e., performed without specific training) behaviours of an “ideal dog” for a given AAS task in relation to basic emotional tendencies [16,42]. Alternatively, tests in dogs may include cognitive measures rather than temperamental characteristics, as individual differences in cognition explain variance in working dog success [43]. For cats, it remains uncertain whether the Fe-BARQ comprehensively captures all dimensions deemed useful for AAS. Thus, forthcoming studies should ascertain additional measures or dimensions, such as personality assessments, caregiver attachment, cognitive measures, and medical history, that warrant inclusion in feline AAS evaluation protocols.

Additionally, when using questionnaires for selection purposes, it is imperative to regard them as a proxy for behaviours. Integrating questionnaire assessments with behavioural evaluations in future research would enhance comprehension, as previous studies have shown discrepancies between survey ratings and video [44] or smart collar [45] recorded behavioural measures in cats. In dogs, not only C-BARQ ratings [46] but also behavioural assessments derived from behavioural examinations and simulated AAS sessions, incorporating diverse features and potential stressors, are utilised for selection and well-being assessments [47], including the identification of dogs’ behavioural prerequisites for AAS. Also in cats, combining caregiver-rated questionnaires with behavioural ratings may advance questionnaire development in relation to cat well-being evaluation.

Importantly, our study did not assess how cats may cope with features of AAS contexts—neither their coping strategies with potential stressors nor their enjoyment of specific aspects. Hence, while our findings provide a first step in describing behavioural profile differences, future research is warranted to explore its relevance in developing potential selection criteria for cats participating in AAS. Specifically, we did not directly evaluate the “suitability” of cats for AAS, neither in terms of their own well-being nor the well-being of others (e.g., human recipients in AAS, cat caregivers, other AAS-engaged cats). Consequently, our findings should be regarded as descriptive, describing behavioural profile differences utilizing the Fe-BARQ as an assessment tool. Future studies should further explore its prescriptive use, assessing the impact on human and animal well-being.

Overall, our study contributes to the ongoing discourse surrounding the above critical themes. We refrain from drawing definitive conclusions regarding the suitability of cats based solely on our findings. Nonetheless, there is potential for further investigation into whether these behavioural profiles correlate with cats’ capacities to navigate challenges commonly linked with specific types of AAS. However, the evolving nature of our understanding necessitates caution in prematurely affirming such correlations. Our work underscores the importance of future research endeavours aimed at elucidating these complex relationships.

### 4.3. Limitations

First, we recognise that the number of AAS cats was very small. However, while the small sample size may lead us to be conservative about the extent to which these behavioural profiles are representative of the wider population of AAS cats, it is important to consider that doing so was not the aim of the study. Instead, we analysed whether group differences existed between AAS and non-AAS cats using a statistical analysis approach that was chosen specifically because of its ability to estimate these differences despite differences in group size. Given that cats have only recently begun to be considered as therapy animals, the unequal group sizes reflect a real-world condition suggesting that the findings are ecologically valid. And while unequal group sizes can affect statistical power, they do not inherently invalidate the results in this case. The fact that significant individual differences were observed despite unequal group sizes suggests that the effect size is in fact sufficiently robust, as evidenced by our effects ranging from small to medium effect sizes. As we were particularly interested in behavioural variation between groups to facilitate meaningful comparisons between these populations, we aimed to preserve crucial between-group variances by maximizing heterogeneity rather than matching the non-AAS cats to the AAS cats. As the number of cats engaged in AAS increases, future research should be conducted sampling larger groups of AAS cats.

A second limitations is that our study did not allow for detecting differences in cats’ enjoyment of AAS features or their ability to cope with potential stressors. Future research would benefit from utilizing methods, such as video-recorded behavioural observations [44], physiological measures [45,48], or assessments of personality and the cat–human bond [49], to evaluate and compare Fe-BARQ scores. These approaches can help predict cats’ suitability for AAS, indicating the enhancement of both human and animal well-being as a result of AAS [20,48,49].

Third, our findings may be attributed to confounding effects regarding the Fe-BARQ’s applicability to AAS, as caregivers may have rated the Fe-BARQ not based on typical day-to-day events. Instead of reflecting genuine cat behavioural differences, observed differences could stem from caregiver variations in interpreting these behaviours or the contexts in which they occur. It is noteworthy that the Fe-BARQ is designed to assess “a cat’s typical responses to ordinary, day-to-day events and stimuli”, explicitly prompting caregivers to rate scales based on such events. However, the engagement in AAS may extend beyond typical day-to-day events, potentially leading to caregiver confusion in interpreting the questionnaire. Insight into this issue may be enhanced by incorporating an understanding of caregivers’ attachment to their cats, which can be assessed using tools such as the Lexington Attachment to Pets Scale (LAPS [50]).

Fourth, caregivers of AAS and non-AAS cats may differ not only in their interpretation of the Fe-BARQ but also in personal attributes, such as their attachment to their cat. Those engaging in AAS with their cat may project specific traits onto them compared to those who do not. For instance, caregivers who are emotionally attached to their cat are more inclined to participate in AAS with their cat [51], potentially influencing Fe-BARQ interpretation and causing unintended bias. Likewise, the social desirability of respondents to present the profile of AAS cats as “desirable behavioural profiles” might be at stake.

Last, our method did not differentiate between various aspects of AAS. Distinctions, such as between resident AAS (cats living where AAS occur) and AAS involving cat relocation by caregivers, could impact cats differently. Hence, behavioural profiles of cats in specific AAS types may vary. Although our study did not aim to address this sensitivity, future research could benefit from delineating between types of AAS and cat differences within them. In doing so, future research would benefit from applying uniform and updated terminology to distinguish between types of AAS [3,27].

## 5. Conclusions

This study is the first to evidence behavioural profile difference between cats engaged in AAS and those not involved. We revealed distinct behavioural profiles, particularly in sociability, attention-seeking behaviour, predatory behaviour, and resistance to restraint. Notably, desirable characteristics identified in AAS dogs appear to also manifest in AAS cats. Second, we demonstrated the feasibility of using the Fe-BARQ, a caregiver-rated questionnaire, to assess these behavioural differences. The utilised Flemish translation of the Fe-BARQ facilitates broader applicability and validation across populations. Our results may hold meaningful implications for future studies, suggesting the incorporation of Fe-BARQ ratings in identifying AAS cat suitability, promoting animal and human well-being. Our study serves as an exploratory endeavour, and future research developing such applications should complement Fe-BARQ assessments with behavioural observations or physiological measures to explore its prescriptive utility in AAS cat assessment and AAS design.

## Figures and Tables

**Table 1:** Subscales and their Cronbach’s alpha in the original Fe-BARQ [28] and in our sample.

Subscale	α _original_	α _our sample_
Playfulness/activity	0.923	0.914
Sociability	0.938	0.946
Directed calls/vocalizations	0.774	0.769
Purring	0.830	0.746
Attention-seeking	0.794	0.790
Sociability with cats	0.908	0.863
Stranger-directed aggression	0.885	0.914
Touch sensitivity/caregiver-directed aggression	0.756	0.747
Resistance to restraint	0.875	0.763
Familiar cat aggression	0.876	0.847
Dog aggression	0.878	0.667
Fear of unfamiliar dogs/cats	0.780	0.820
Fear of novelty	0.822	0.815
Separation-related behaviour	0.859	0.822
Trainability	0.664	0.767
Predatory behaviour	0.809	0.780
Prey interest	0.760	0.737
Location preferences for resting/sleeping *	0.501	0.301
Excessive/compulsive self-grooming *	0.712	0.609
Other compulsive behaviours *	0.617	0.597
Inappropriate elimination	0.675	0.750
Elimination preferences	0.612	0.742
Crepuscular activity *	0.615	0.504

*N* = 474 cats. * Subscales that were excluded from our analyses.

**Table 2:** Results of two-tailed, non-matched samples Mann–Whitney U-tests * examining group differences based on whether cats engaged in at least one type of AAS.

Subscale	Mdn _AAS cats_	Mdn _non-AAS cats_	*p* Value
Playfulness/activity	2.82	2.23	0.064
Sociability	2.93	1.86	0.048
Directed calls/vocalizations	3.00	3.00	0.759
Purring	3.00	3.00	0.439
Attention-seeking	3.50	3.00	0.042
Sociability with cats	2.00	0.67	0.036
Stranger-directed aggression	0.00	0.00	0.995
Touch sensitivity/caregiver-directed aggression	0.00	0.25	0.178
Resistance to restraint	0.00	0.67	0.029
Familiar cat aggression	0.00	0.50	0.080
Dog aggression	0.33	0.40	0.419
Fear of unfamiliar dogs/cats	0.50	1.75	0.419
Fear of novelty	0.50	1.00	0.266
Separation-related behaviour	0.25	0.33	0.646
Trainability	3.00	2.67	0.276
Predatory behaviour	4.00	2.33	0.006
Prey interest	3.00	2.50	0.636
Inappropriate elimination	0.00	0.00	0.951
Elimination preferences	0.00	0.00	0.591

*N* = 474 cats. * Accounted for unequal group size and variances of independent groups.

## Data Availability

The raw data supporting the conclusions of this article will be made available by the authors on request.
